# Initiating undergraduate medical students into communities of research practise: what do supervisors recommend?

**DOI:** 10.1186/1472-6920-10-83

**Published:** 2010-11-19

**Authors:** Margaret MacDougall, Simon C Riley

**Affiliations:** 1 Public Health Sciences Section, Division of Community Health Sciences, University of Edinburgh Medical School, Teviot Place, Edinburgh, EH8 9AG, UK; 2 Centre for Reproductive Biology, Queen's Medical Research Institute, University of Edinburgh, 47 Little France Crescent, Edinburgh EH16 4TJ, UK

## Abstract

**Background:**

Much has been written in the educational literature on the value of communities of practise in enhancing student learning. Here, we take the experience of senior undergraduate medical students involved in short-term research as a member of a team as a paradigm for learning in a community of practise. Based on feedback from experienced supervisors, we offer recommendations for initiating students into the research culture of their team. In so doing, we endeavour to create a bridge between theory and practise through disseminating advice on good supervisory practise, where the supervisor is perceived as an educator responsible for designing the research process to optimize student learning.

**Methods:**

Using the questionnaire design tool SurveyMonkey and comprehensive lists of contact details of staff who had supervised research projects at the University of Edinburgh during 1995 - 2008, current and previous supervisors were invited to recommend procedures which they had found successful in initiating students into the research culture of a team. Text responses were then coded in the form of derivative recommendations and categorized under general themes and sub-themes.

**Results:**

Using the chi-square tests of linear trend and association, evidence was found for a positive trend towards more experienced supervisors offering responses (χ^2 ^= 16.833, p < 0.0005, n = 215) while there was a lack of evidence of bias in the gender distribution of respondents (χ^2 ^= 0.482, p = 0.487, n = 203), respectively. A total of 126 codes were extracted from the text responses of 65 respondents. These codes were simplified to form a complete list of 52 recommendations, which were in turn categorized under seven derivative overarching themes, the most highly represented themes being *Connecting the student with others *and *Cultivating self-efficacy in research competence*.

**Conclusions:**

Through the design of a coding frame for supervisor responses, a wealth of ideas has been captured to make communities of research practise effective mediums for undergraduate student learning. The majority of these recommendations are underpinned by educational theory and have the potential to take the learner beyond the stage of initiation to that of integration within their community of research practise.

## Background

It has been recognized that the pursuit of new ventures is an integral part of human existence [1]. As Wenger explains, however, it is when these ventures are pursued effectively with others that participants may gradually assume particular types of practises and types of social interaction which ensure survival in their individual roles and progress towards their respective endpoints. In the current study, we understand this process to be definitive of that which typifies a *community of practise *in contradistinction to *community *in the more generic sense. It is also implicit from the illustrations provided in the literature that from a pedagogical perspective, such communities ought to be characterized by openness - both in terms of membership and in sharing of new ideas, so that existing norms and established ideas and perspectives are perpetually open to negotiation or challenge. Thus, a community of practise is a community within which "Knowledge is created or negotiated through the interactions of the learner with others and the environment" [2].

*Communities of practise *are rarely discussed explicitly within the medical education literature. Nevertheless, the existing illustrations outlined below acknowledge the potential for communities of practise to serve as valuable constituents of effective learning processes in a diversity of learning contexts within Medicine.

(i) *Undergraduates learning professionalism within clinical settings in early years*. In this case, the medical profession is alluded to as a community of practise - an idea which we shall briefly return to later in this paper. In this setting, exposure to positive and negative role models together with socialisation with a range of clinical staff help to shape individual learners' ideas of professionalism in *Medicine *and enhance their sense of identity as emerging clinical professionals [3].

(ii) *Educators sharing good teaching practise*. Here, it is recommended that interested medical and other health educators, the community of (professional) practise, develop a virtual evidence-base as a collaborative tool for negotiating a) good practise in the use of Web 2.0 technology in student learning and b) the content of learning materials for sharing among the community [4].

(iii) *Clerkship case presentations*. Within this context, case presentations are used as a pedagogical tool for enabling undergraduates to engage in clinical discourse about paediatric inpatients. Thereby, they learn the boundaries of such discourse within individual clinical disciplines, together with associated values, goals and ways of thinking. Correspondingly, the professional communities within these disciplines are viewed as communities of practise [5].

(iv) *Pre-registration house officer training*. Here, the ward team is the community of practise and learning as an apprentice is understood to lead to "professional adhesion" within different specialties through "socialisation into attitudes and values" [6].

(v) *Review of research experiences of students ('the novices') enrolled in Masters and PhD programmes in medical education*. In this case, a call is made for good quality medical education research on the development of communities of practise to connect "novices" and "experts" [7]. With reference to "the process of supervising [student] research in a medical education setting" [7] Pugsley emphasizes that:

"Further work is needed to develop the most effective model of supervisory support for supervisors and to promote sharing of good practice." [7]

(vi) *Involving undergraduate medical students in existing communities of research practise and giving undergraduate research scholarship status (McLean, personal correspondence)*. McLean and Howarth raise the concern that "[w]e... owe it to our students to socialise them into communities of practice where the scholarship of discovery is valued" [8].

In this study, we consider the specific paradigm of undergraduate medical students being initiated into the culture of research teams and as such, becoming recognized members of communities of (research) practise. While recognizing that Pugsley's comments need not be restricted to the field of medical education, we take a pragmatic approach to the challenges raised by the authors in illustrations (v) and (vi) through highlighting principles of good supervisory practise.

In viewing the research team as a community of practise, we recognize the role of the disciplinary context [9] (especially the culture) in shaping the nature of research within that community. Also, we assume that students as learners adhere to a social constructivist epistemology according to which knowledge is not sacrosanct but continually open to fresh perspectives and modification based on new experiences and "shared understandings" [10].

Moreover, we see communities of research practise as having the potential to make Brew's notion of knowledge "as a product of communication and negotiation" a reality. According to her theory of learning, these communities should as such make the relationship between research and learning a more "intimate" one [11]. Consequently, the findings of the current study should challenge the idea that research is a manipulative process used by staff for their own profit to the detriment of student learning or that the research-teaching nexus is a myth [12]. Each of these ideas are by-products of a more traditional model both of the relationship between teaching and research and of the likely participants in these processes [11].

In some cases, undergraduate student projects will at least partially involve participation in and examination of the findings of a clinical audit up to the stage of comparing collected data with set guidelines or standards. It has been suggested that research is "about obtaining new knowledge" (including that of "best practice") while *by contrast*, "Clinical audit is about finding out if best practise is being followed" and may involve identifying "areas where the research evidence is lacking" [13]. However, it can also be argued that so defined, audit is about obtaining new knowledge ("finding out") and as such, constitutes a type of research, albeit one which requires to be carefully defined. Thus, it will be assumed here that recommendations for good supervisory practise in respect of research projects also encompass areas of the projects involving audit.

The authors' motivation for disseminating good principles of supervision in initiating students into communities of research practise is learning-focused. In particular, we intend that the student be validated as a partner in the construction of knowledge within their community of practise. For knowledge construction to be valuable to the community, it need not be confined to new research areas. The student researcher also has the capacity to provide new ways of seeing and defining what is already known within territory with which the rest of the research community is already familiar [14]. In either sense, knowledge construction involves intentionality [15] and as such also contributes to a deeper learning experience for the student. Thus, the pedagogical underpinning of this study is not that of investigating exactitude in adherence to a formulaic model of a community of practise in antipathy with the more open-ended model recognised in the literature [1]. Rather, taking the research team to be an example of a community of practise, we intend to provide a knowledge base of recommendations to ensure that the novice researcher has rights of passage to the culture of the research team and is therefore able to use socialisation within that team as a positive learning experience [7].

## Methods

The particular type of student research considered in this study is encountered within the 4^th ^year student selected component (SSC4) at the University of Edinburgh, Scotland. This mandatory programme involves clinically related projects which typically take place over a 14-week period, leading to the submission of a project report of about 3,000 words. These projects are also recognized explicitly within the course materials as having the potential to increase student research skills and as counting towards summative assessment, with the project mark being assigned a weight of 14% in deciding end-of-year marks.

Our target population consists of all staff who acted as SSC4 supervisors for the MBChB programme at the University of Edinburgh during the period 1995 - 2008 as specified in existing lists provided by the SSC Secretary. This population comprises that of a more extensive survey-based study involving an investigation of research-teaching linkages. All supervisors were briefed in advance by email of this larger study and directed to the corresponding link on the Higher Education Academy website for further details. The question which is of relevance to the current study was integrated into the questionnaire for the larger study using the questionnaire design tool SurveyMonkey (Professional Version).

The study question was presented as follows, together with the response categories 'Yes' and 'No':

"Can you recommend any procedures for other supervisors which you have found successful in initiating students into the research culture of a team?"

Using skip logic within the SurveyMonkey system, those supervisors who responded affirmatively to the above question were then prompted to share their recommendations in free text form.

The remaining questions within the original questionnaire were mainly designed to assess the utility of the Edinburgh SSC4 experience in enhancing the research-teaching nexus.

### Qualitative Analysis

The software system Atlas.ti (Version 6.0) was used for coding recommendations within responses from individual supervisors, for keeping a record of mappings between the original supervisor comments and the codes allocated to them and for text searching. An in vivo coding frame was developed independently by each of the authors based on a scrutiny of all of the free text responses to the prompt for recommendations. This process included mapping individual and merged short phrases offered by respondents into more coherent individual recommendations (the codes). The final version of this coding frame, comprising 52 codes, seven derivative overarching themes in the form of general recommendations, together with four sub-themes was agreed by an iterative process involving discussions between the authors and, in some cases, conversion of in vivo codes to axial codes through grouping of related components of responses [16].

The authors worked independently of a checklist of criteria for data interpretation and without assuming the objective existence of a *true *coding framework for the study data. Furthermore, due to the subjective nature of the task, it was considered untenable for a single researcher either to construct a valid coding frame in isolation or to choose from a pre-defined fixed list of response categories in identifying themes and recommendations. Otherwise, there would have been a clear call to assess inter-rater reliability of findings. Rather, in keeping with established practise in qualitative research [17], the above iterative approach was viewed as an effective means of refining the emerging coding frame based on fresh insights gleaned from the independent perspectives of the authors.

Since respondents were already exposed in part to the conceptual framework behind this particular study through the wording of the survey question, the need for axial codes was less extensive than in other qualitative studies. Hence, relative to Charmaz's notion of constructivist grounded theory [18], the grounded theory approach assumed within this study may be regarded as semi-constructivist in that during the identification of codes there was a lesser need to "confer meaning" [18] on the already existing data.

### Statistical analysis

For the purpose of statistical analysis, the numbers of students supervised by any one supervisor to the stage of project completion were assigned to the categories '1', '2 - 5', '6 - 10' and '> 10'. These categories were in turn used to represent levels of supervisory experience.

Using the chi-square test of linear trend, levels of supervisory experience were compared across those who responded 'Yes' to the study question and provided recommendations and those who responded 'No' and therefore were not prompted to contribute. The chi-square test of association was also used to test for a difference in gender across respondents who did and did not contribute recommendations for good supervisory practise in the above sense. These procedures were performed using the software package SPSS (Version 14.0) and a significance level of 0.05 was assumed for hypothesis testing.

## Results and Discussion

Of the 217 respondents from the larger study, 67 (30.9%) responded 'yes' to the above question, and all but two of these particular respondents provided one or, more often, several recommendations on practise they had found useful. The remaining two supervisors provided observations only. These supervisors were excluded from the study, as these observations lacked adequate clarity to formulate codes representative of action points for supervisory practise. The above two supervisors comprised one male and one female who had both supervised 2 - 5 students. A total of 127 recommendations were collected in text form from the 65 remaining respondents, who represented a wide range of clinical specialties (98 in total). Of these recommendations, 126 were coded using a classificatory list of derived recommendations and in turn, allocated to a theme, so as to form a coding frame.

The associated distributions of supervisors according to level of experience and whether recommendation(s) were offered are summarized in Table 1. These results reveal a monotonic trend towards higher proportions of contributors relative to non-contributors as number of students supervised increases. The chi-square test of linear trend confirmed that this trend was statistically significant (χ^2 ^= 16.833, p < 0.0005, n = 215). Recommendations were collected from 44 (33.6%) of a total of 131 male survey respondents, 20 (27.8%) out of a total of 72 female survey respondents and 11 supervisors who withheld their gender status. For the available data, there was no significant difference between proportions of males and females from whom recommendations were received to inform this study (χ^2 ^= 0.482, p = 0.487, n = 203), thus confirming a lack of statistical evidence for bias in the gender distribution of respondents.

**Table 1:** Distributions of frequency and percentage of respondents providing recommendations according to number of students supervised

No. of students supervised	Recommendations provided?		Total
	No	Yes*	
1	32 84.2%	6 15.8%	38 100.0%
			
2 - 5	71 75.5%	23 24.5%	94 100.0%
			
6 - 10	31 68.9%	14 31.1%	45 100.0%
			
> 10	16 42.1%	22 57.9%	38 100.0%

**Total**	150 69.8%	65 30.2%	215 100.0%

*Total does not include two supervisors who were excluded on grounds of lack of clarity

The seven overarching themes which emerged from the coding process together with the number of respondent supervisors who contributed to these themes are provided in Table 2.

**Table 2:** Seven overarching emergent themes and four sub-themes (italics) relating to supervisor recommendations for initiating undergraduate medical students into communities of research practise

Theme	No. of corresponding quotations within supervisor responses
Planning the research for the student	42
Preparing the student for the research	40
- *Clearly defining roles, rights of ownership and expectations*	
- *Getting to know the student*	
- *Engaging the student in prior learning*	
- *Getting the student started*	
Connecting the student with others	57
Cultivating a sense of accountability	41
Fostering a holistic perspective of the subject area(s)	24
Cultivating self-efficacy in research competence	46
Working with the student	31

The complete list of derivative codes in the form of recommendations and the coding frame for these recommendations, indicating their position relative to over-arching themes and sub-themes are also available (see Appendices A and B, provided as Additional files 1 and 2, respectively).

The recommendation "To supervise well needs some existing research/audit experience" was not allocated to a theme. Instead, we offer it here as a preliminary word of caution and baseline principle to programme organizers in the recruitment of good supervisors and to staff considering taking on the role of a supervisor.

Each of the remaining supervisor recommendations discussed below is recognized under what we consider to be the most compelling theme.

### Planning the research for the student

The recognition that planning the project amounted to planning primarily *for the student *was expressed in a number of ways. For example, the need to plan well ahead or from the outset for the student was evident in various responses. These included the recommendation to seek to remove potential logistical problems and to prepare a structured schedule early on to cover the duration of the project. In several cases it was also evident that the supervisor was to be a key player in designing the project. This was expressed in terms of the need for the supervisor to have clear goals from the outset and to set clear outcomes.

These examples were complemented by recommendations which allowed for a more interactive element in the supervisor-student relationship. Examples of the latter sort included recommending that supervisors discuss options early on, highlight "the importance of... specificity" in discussions on "the research question" and agree on research questions together with the protocol (including how to recruit patients) *several weeks *before the start of the SSC4 period. These recommendations all carry the intention of planning to ensure the goals for the student are achievable. This intention resurfaced more explicitly through other recommendations involving supervisor responsibilities, including planning the project to ensure early successes based on their own knowledge of what has worked in earlier research or research-related activities and setting or agreeing on realistic goals.

A further recommendation was that of tailoring the project in line with the research interests of the group which the student would be identified with during their period of supervision. Three concrete examples of this approach emerged, the first of which was getting the student involved in a prospective study (particularly where this might include or constitute a pilot study). This example has the particular advantage of allowing the student to cover previously uncharted territory of direct value to other team members. Related ideas extended to students producing information leaflets and FAQ sheets on the basis of their findings - activities which were perceived to be beneficial not only to the student, but to other departmental members, thus enhancing the student's sense of community. Secondly and more generally, supervisors were advised to ensure that the student project was embedded within a larger ongoing project which the research team were already involved in. The latter recommendation in particular was viewed by one respondent as having the spin-off effects of making the team more enthusiastic about the research and better placed to share their expertise with the student. Thirdly, it was suggested that the student be afforded the opportunity to be involved in the "development phase of the project".

Supervisor planning activities were not exclusively recognized as taking place prior to the commencement of the project, however. For instance, supervisors were advised to "be on the lookout for potential problems which the student may encounter with notes reviews."

Planning activities were also recognized as being of relevance to elements of the student research experience which were not integral to the requirements of the project. In particular, supervisors with high expectations in terms of student presentations at national or international meetings were advised to plan for funding to support the students' attendance at these meetings.

### Preparing the student for the research

#### Clearly defining roles, rights of ownership and expectations

Within the context of short research projects, undergraduate students remain "scholars in training" [8] and supervisors need to remain sensitive to possible naivety in students' understanding of the research experience. Recommendations consistent with this perspective included highlighting the potential setbacks, obstacles and contradictions associated with the process of doing research, while recognizing the contrast with other forms of learning. Specific reference was made to hypotheses not being confirmed and experiments failing. Furthermore, supervisors were advised to brief the student on "what is expected" during their "attachment", clarify the student's role within the team and explain the role of the researcher in a laboratory setting. The value of foresight *beyond *the stage at which the student must submit their project report was also recognized through the recommendations that supervisors should clarify rights of ownership of data and anticipated roles in preparing work for publication.

#### Getting to know the student

Further recommendations for proactive supervisory activities included the suggestion that supervisors familiarise themselves with curriculum timetables at the level of the individual student. Likewise, a recommendation derived from the responses was that of exploring and identifying what makes a given student challenging to supervise. Such practise would clearly serve as useful preparatory work for supervisors aspiring to the additional recommendation that they "Design a bespoke formal training programme for each student." Any application of the latter recommendation could be particularly effective if programme organizers ensured that student training in key research skills was situated within the experience of research. This ought to include training with professional feedback leading to the design of a coherent project proposal with potentially achievable and measurable outcomes.

Not all deficits in beneficial prior learning can be dealt with through mandatory training, however. The study findings revealed that prior experience of laboratory research, such as through vacation projects or relevant intercalated honours course experience was viewed as favourable for laboratory-based projects. Supervisors ought to be aware, therefore, of the scope of small summer projects and similar activities for recruiting students in earlier years into preliminary training in for example, laboratory techniques or instrumentation, and should advertise suitable opportunities accordingly.

#### Engaging the student in prior learning

The majority of recommendations under the current over-arching theme, *Preparing the student for the research*, fell under the sub-theme *Engaging the student in prior learning*. For example, the idea of supervisor-led training resurfaced through the recommendation that supervisors provide an introduction to the research topic.

Further recommended activities included involving the student in background reading, through for example, provision of "a good introductory book on research methods," putting the student "in touch with all specialists for all necessary training" and encouraging the student to sign up for relevant courses. Additionally, it was advised that supervisors should encourage their students to carry out a short literature review with a view to them becoming well-versed in their subject area prior to performing "basic techniques." Literature reviews may be seen as an effective means of enabling the student to learn the language and the research history of the discipline, thus empowering them to negotiate project objectives and emphases, both with their supervisors and other members of the project team. Consequently, literature reviews may allow the student to assume project ownership to a greater extent, thus enabling them to engage more effectively with social learning as a non-peripheral member of their community of practise.

This more self-directed approach to student learning was also apparent within the context of the student coming to terms with the realities of the research process through a preliminary risk analysis. In particular, it was advised in the case of clinical projects that the student should be encouraged to visit the unit early on and identify any practical problems they might encounter during the project. Such practise could serve the student well in protecting them from an over-optimistic approach to the efficiency with which they can hope to achieve the deliverables of their research project, particularly at the early stages, where for example, the problem of delays in obtaining essential patient data can prove frustrating.

#### Getting the student started

The recommendation from the response data of getting the student "started" prior to the official start date for their project and in other cases "early" should be seen as fundamental. Such practise is clearly intended to guard against the undesirable event of a student being left 'out in the cold' at the start of their research period through poor planning on the part of the supervisor.

### Connecting the student with others

The current theme assumes the highest number of supervisor recommendations in this study. This is important to note on considering the importance of social connections to the *negotiation of meaning*, an activity which is considered central to participation in a community of practise [1]. Thus, we recognize a meeting of minds in what supervisors recommend as research practitioners and what is supported within the learning theory literature as typifying engagement within a community of practise.

Within this particular study, negotiation of meaning may for example, relate to the interpretation of what constitutes clinical research within a given field or more specifically, the interpretation of research findings. While supervisors proposed a wide range of recommendations for including the research student in social connections, a popular recommendation was that of personally introducing the student to, or involving the student in, the research team early on. The need was also recognized for a "key player" or "main contact" to be identified from the research team as a reference point for the student, including when obtaining data. Indeed, one supervisor recommended that such a contact should be "available on a daily basis". Additionally, inclusion at the project planning stage of persons previously involved in similar research was seen to be desirable, as was the inclusion of a clinical research fellow or junior doctor in the research team.

The need for reinforcement of similar connections was expressed through the recommendations of including other team members in review meetings and meeting regularly as a team. In the latter case, the interpretation of 'regularly' might vary according to the nature of the project; for example, in the case of laboratory-based research, weekly meetings were suggested.

At a slightly less formal level, it was suggested that the student be allowed to shadow a team member and that they should be introduced to all members of a departmental meeting, not just team members.

Suitable contacts were not restricted to staff, however. References to other students (including previous SSC4 students and postgraduate students) as contacts or persons to be involved in the projects were made by five supervisors. Practical steps for supervisors to connect their student with peers in this way included assigning a "previous student" as a mentor or tutor to the new student in a large ongoing project and allocating a room to the new student with other students, including postgraduate research students. The strategy of new students connecting with previous students in the form of mentors within the framework of a large project is an attractive one in the light of claims that knowledge shared between peers and "near-peers" is circulated extremely "rapidly" and "effectively" [19]. Such activities should also be seen as particularly advantageous to all participating students where it might be the case that supervisors are unable to assume the role of expert for the specific area the new student has chosen to research. As Brew notes:

"The process of passing on notes and findings to the next group of students develops the sense of a community of experts" [20].

This result is undoubtedly due to the sense of continuity, orientation and optimism which arises from further developing previous projects which have already been recognized as successful. The design of an evolving project with meaningful stages for successive students to disembark is also likely to strengthen the resilience of the community of practise as a whole to the discontinuities in learning which can arise through short-term participation by research students. This is clearly important when one considers that within a community of practise, practises are understood to "evolve as shared histories of learning" [1].

Recommendations falling under the current theme also involved relations extending beyond the departmental level. This was evident within the context of empowering the student to take up educational opportunities, including:

a) *consulting the statistician early on *- a recommendation which could be suitably applied to consultations involving the supervisor, the student or both the supervisor and the student

and

b) encouraging the student to interact with patients and their families.

If, as in this study, we think of the community of practise as the research team to which the student belongs, then these illustrations extend possible connections to interactions with persons who are outside that community but who are also in some sense connected with it in terms of how it functions. This, together with findings later in this paper, confirms that supervisors are open to student researchers feeding knowledge back to the community of research practise and supports the model of communities of practise as "not just internal... [but] histories of articulation with the rest of the world" [1]. In this setting, the student can act as broker [1] between their external contacts and the members within their community of practise with regards to conveying appropriate types of professional practise recommended by trained specialists. This sharing of revelations can have a formative effect within a community of research practise through transforming the meaning of research practise and output, thus illustrating the capacity of brokering to reify a research student's sense of indispensability within their community of practise. Furthermore, the reconciliation of knowledge across boundaries gives rise to a higher level of learning on the part of the student through deeper engagement [1].

Recognition of the need for such activities is also grounded on the principle that the locality of a community of practise is defined by the existing perspectives on practise to which it adheres and the resultant need for cross-community dynamics, possibly within a constellation of communities of practise [1]. This fundamental characteristic points to the need for greater clarity and specificity in the usage of the term *community of practise *than is forthcoming from referring, as in [3], to *the medical profession *in general.

Interactions with patients also have the potential to enable students to engage with issues relating to professional conduct in a participatory manner, particularly with respect to the patient's right to withhold information requested for research purposes and the use of informed consent [21]. This has the bonus of enhancing a student's sense of self-efficacy in terms of appropriate behaviour for a competent researcher.

### Cultivating a sense of accountability

Supervisors were advised to meet regularly (it was suggested monthly) to assess progress and review short-term goals. They were also advised to consider shadowing the student at discussion and planning meetings. Shadowing the student in this way, for example, in consultations with a statistician, has the potential to enlighten the supervisor on extensive advice provided to the student and corresponding educational resources which they have been directed to. As such, the above strategy can alert the supervisor to reasonable obligations on the student's part which the supervisor may not otherwise have been aware of.

The idea of student accountability extended to administrative roles, with supervisors being recommended to encourage their students to develop skills in minute taking during student-supervisor meetings and larger research team meetings.

Conceptually speaking, one would expect the activity of *encouraging a sense of project ownership *to be exemplary of supervisor behaviour in keeping with the current theme. However, this activity did not come through sufficiently strongly in the supervisor recommendations even to constitute a sub-theme. This may be linked to the fact that self-proposed projects, although recognized within the Edinburgh SSC4 programme, are more the exception than the norm. Students are more likely to choose a project topic and corresponding supervisor(s) from a comprehensive list provided by the SSC Director [22]. Here, scope for autonomy arises from negotiation between the supervisor and the student regarding project objectives within the framework of pre-existing boundaries. The above tendency for restricted autonomy undoubtedly exists for good reasons, including students' lack of prior research experience and the resultant need for extensive training in research design.

### Fostering a holistic perspective of the subject area(s)

Supervisors were advised to ensure that the student was clear about the value which their contribution could make to current knowledge and practise on a wider scale. An emphasis was placed on the importance of students being included in routine departmental activities, such as lab meetings, seminars or clinical meetings, rounds or sessions, where the agenda need not be ostensibly relevant to the interests of the project. In some cases, supervisor recommendations reflected that this might simply involve advising the student of the existence of such activities.

More specifically, however, recommendations for involvement of the student in meetings also covered multidisciplinary team meetings, thus offering the potential for broadening the student's learning perspective further.

It is noteworthy that the current theme should have emerged from the supervisor responses to the study question, as this theme resonates well with Wegner's observation that "An important aspect of the work of any community of practice is to create a picture of the broader context in which its practice is located " [1].

### Cultivating self-efficacy in research competence

Supervisor enthusiasm about the project, the subject area(s) of the project or some other non-specified but presumably relevant area was recognized as important. The value of optimism was also recognized through the recommendation that supervisors inform their students of previous successes. This strategy might encompass the recommendation, derived from the response data, of getting the student to read the reports of previous students. The perspective represented here of previous students as role model researchers is very much in keeping with that of addressing undergraduate medical student concerns that the research task is "beyond them". In the latter case, the use of a poster exhibition displaying research findings from a previous cohort has proven to be an effective strategy [21].

The idea of recognizing the capacity for students to take on the role of researcher was also evident in the recommendations that supervisors take their students' ideas seriously and that they cultivate a questioning attitude. These recommendations place value on the student voice, both in research design and in the interpretation of past and present findings. As such, they reflect a receptive attitude towards deviant opinions or findings, thus acknowledging the fundamental need of allowing the student to challenge current thinking and practise within the research team. Since the very life of the research team as a community of practise depends on change [19], it is inconceivable that a student can be initiated into that community without recognizing realistic openings for new perspectives or unexpected findings.

Supervisors were also advised to discuss with their students future opportunities, such as presentations at major conferences, which might have a powerful influence on students' career profiles and to get them to present their "findings" or "data" at one of the group seminars. The recognition of recommendations of this type as supportive of initiation into a community of practise is entirely consistent with findings elsewhere in relation to undergraduate student research in science, where supervisors recognized that "attending conferences helped students to... view themselves as part of the [entire] scientific community" [23]. However, the activities recommended here can also empower students to articulate their own interpretations based on their research within a context where long-standing researchers are available to fine-tune or validate these interpretations and indeed, to offer divergent explanations. Consequently, they must also be seen as highly supportive of Wenger's process of *reification of meaning *whereby the learner is empowered to recognize their refined ideas as constituents of their discipline which are worthy of recognition in shaping their community of practise [1]. Moreover, students are exposed to appropriate "forms of discourse" [5] to enable these ideas to gain greater acceptance within their community of practise through use of rhetoric to convey competence and credibility.

### Working with the student

The critical need for confirmation of clarity on the student's part regarding the project proposal was recognized under the recommendation of getting the student "to sketch out their final report before they start." The value of this recommendation cannot be over-emphasized in giving the student a sense of direction for preparing a clear and coherent project plan prior to seeking statistical advice on how to analyse their data.

The above approach to mobilizing the student was counterbalanced, however, by the recommendation that the supervisor be "actively involved throughout". The latter recommendation is entirely in keeping with the view that educators should represent their communities of practise as "active practitioner[s]" to communicate a "lived authenticity" to the subject matter. This approach is understood to lead to more purposeful learning [1]. Within the context of communities of practise, such as those referred to in this study, this benefit can take the form of empowering the learner to imagine what they could be beyond the restrictions of their curriculum.

The suggestions "Make yourself accessible" and "take into account absence of research experience by allowing lots of time in your schedule to provide guidance" were merged under a common derivative code. The strategies which they represent have great potential value in enhancing self-efficacy in research competence and serve as a cautionary note against taking on too many students within any given year.

The remaining 12 codes of relevance to the current theme fell first and foremost under one of the themes *Planning the research for the student*, *Cultivating self-efficacy in research competence *and *Cultivating a sense of accountability *or one of the sub-themes *Engaging the student in prior learning *and *Getting the student started *of the theme *Preparing the student for the research*.

More generally, the activities recognized under this theme as a whole (Additional file 2) are an essential basis for providing feedback on performance throughout the project period and as such, ought to have a formative effective in developing the student as a researcher.

### Strengths and Limitations

The proportion (30.9%) of respondents who declared that they were able to offer recommendations for future supervisors in relation to initiating students into the research culture of a team was surprisingly low. Nevertheless, the tendency, confirmed through statistical hypothesis testing, for recommendations to come from more experienced supervisors may have served to enhance the quality of the recommendations made and would certainly have explained their plenitude, there being 52 derived codes allocated to themes, based on a total of 126 recommendations. It was also heartening to verify that there was an absence of statistical evidence for a bias in the gender distribution of respondents.

Research opportunities are a common feature of undergraduate medical programmes on an international scale. Thus, the supervisor recommendations forthcoming from this study ought to be recognized as transferable well beyond the confines of SSC programmes within the UK. However, sources of diversity exist across medical school undergraduate research programmes which may serve to render some of the recommendations from this study less relevant. We consider here a few pertinent examples.

As in the case of vacation research projects, research may be extra-curricular [24]. Within these contexts, a higher proportion of students may feel more motivated to engage in research and less driven on extrinsic grounds. Research programmes may also differ in terms of eligibility requirements for student participants. For example, places for the Norwegian Medical Student Research Programme [25] are very restricted and competitive, with 53/580 (9.1%) of medical students participating in the programme across all four Schools in any one year. In such cases, students may require less input from supervisors to enhance self-efficacy. They may also show more initiative in connecting with others and have a higher sense of personal accountability.

Opportunities for longer-term programmes for undergraduate research also exist. Examples of medical syllabi which facilitate more long-term supervised research projects include the above Norwegian Programme (which runs for two years, with the first year in parallel with the undergraduate medical curriculum), the mandatory undergraduate research programmes for 2^nd ^and 6^th ^year students at the United Arab Emirates University (UAEU) [8], the mandatory programme provided in Years 1 - 4 under the Scholarly Project Initiative at the University of Pittsburgh, Pennsylvania [26] and required third-year research experiences of up to 21 weeks at Mayo Medical School [27]. US fellowship programmes also exist where successful applicants take one or two years out of their undergraduate degree programmes to pursue scientific or clinical research [8,28], not to mention joint MD - PhD programmes involving time out of at least 3 years from undergraduate degree programmes [28].

Within such programmes, opportunities for situated learning in research methodology will be more abundant. For example, the Norwegian, UAEU and University of Pittsburgh programmes offer considerable scope for extensive preparatory training in research methods and a rigorous review process for research proposals prepared by the students. Likewise, Mayo Medical School require a review process to take place through their research co-ordinating committee [27]. Based on the constructive feedback forthcoming from these processes there is likely to be a lesser need for an emphasis to be placed on some of the recommendations for supervisors identified under the overarching themes *Planning the research for the student *and *Preparing the student for the research*. Also, even within existing UK SSC programmes, the amount of opportunities for undergraduate students to participate in research may vary according to the research ethos of the School [22] and there may therefore be a corresponding need to be selective in matching supervisor recommendations from this study to specific types of project within any given programme.

Typically in the UK, as with the projects considered here, the time allocated to research projects is very limited relative to the curriculum as a whole. These time restrictions set a boundary around the community of practise which the new researcher experiences, leading to divergence between the community of research practise as perceived by the student through the lens of their curriculum and as realized in the workplace. This is evident both in terms of breadth and depth of interactions and contribution to knowledge construction, including publication of research findings.

This raises the issue of whether the identity of the researcher ought to be cultivated and sustained within undergraduate medical curricula through initiating students into communities of research practise in pre-clinical years and developing curricula to facilitate extension of the project work in subsequent years.

## Conclusions

Through the design of a coding frame for supervisor responses, a wealth of recommendations has been captured to enable supervisors to make communities of research practise effective mediums for undergraduate student learning. The majority of these recommendations are underpinned by educational theory and have the capacity to promote a learning context involving mutual engagement - an "essential component" of the type of practise which typifies a community of practise [1].

The associated over-arching themes and sub-themes forthcoming from this study ought to serve as a checklist for supervisors in enhancing research experience. Not all of the derivative codes listed under these themes (Additional files 1 and 2) could be implemented for any one project. However, they offer a rich source of practical ideas on how to implement the above recommendations in a given research setting. If used in these ways, the coding frame for this study should serve to foster a sense of *mutual accountability *between the supervisor and the student, thus helping to keep the community of research practise intact [1]. Moreover, the majority of the recommendations discussed in this paper have the potential to enable the research student not only to be initiated, but also to be *integrated *into the community.

This suggests that, contrary to what has traditionally been assumed regarding the behaviour of newcomers to a community of practise [1], supervisors from this study do not tend to expect research students to operate on the periphery, where "safe" and "casual" forms of practise are dominant. While this more integrative approach may be deeply influenced by the culture of learning in other parts of the undergraduate medical curriculum in senior years, if managed aright, it may also allow exposure to deeper forms of learning. Based on the findings from remaining parts of the survey used in this study, it is intended that the idea of fledgling research students working beyond the periphery will be revisited in a follow-up paper informed by data analysis.

## Competing interests

The authors declare that they have no competing interests.

## Authors' contributions

Both authors have read and approved the final manuscript. MMD: study design, background, literature searching, data collection, qualitative and statistical analysis, interpretation of qualitative and quantitative data, preparation of drafts of paper and final version of paper. SR: literature searching, background, data collection, qualitative analysis, interpretation of qualitative data, provision of recommendations on drafts of paper

## Pre-publication history

The pre-publication history for this paper can be accessed here:

http://www.biomedcentral.com/1472-6920/10/83/prepub

## Supplementary Material

Additional file 1 **Appendix A**. Complete list of derivative codes representative of recommendations for good supervisory practise Click here for file

Additional file 2 **Appendix B**. 7 Overarching themes for good supervisory practise and associated coding frame Click here for file
