# Supplementary material for: Initiating undergraduate medical students into communities of research practise: what do supervisors recommend?
Source: BMC Med Educ. 2010 Nov 19;10:83. doi: 10.1186/1472-6920-10-83 (PMC2998509; doi:10.1186/1472-6920-10-83)
Supplement: Additional file 1 — Appendix A. Complete list of derivative codes representative of recommendations for good supervisory practise [file 1472-6920-10-83-S1.PDF]

Appendix A

Complete list of derivative codes representative of recommendations for good supervisory practise[1]

1. (get student to)[2] read reports of previous students (1)

2. advise them of departmental meetings beyond the scope of their project (2)

3. allocate room with other students, including postgraduate research students; involve junior postgraduate colleagues (3)

4. allow plenty of lead time to consider ideas and carry out background reading; allow time prior to project period for background reading (4)

5. allow the student to immerse themselves in the topic by getting them to carry out a literature review prior to practising basic techniques (2)

6. be enthusiastic about project or subject area, or something else (4)

7. be on the lookout for potential problems which the student may encounter with notes reviews (1)

8. brief student on what is expected during the attachment; clarify the student's role within the team; explain the role of the researcher in a lab setting (5)

9. clarify rights of ownership of data and role in preparing work for publication (1)

10. consult statistician early on (1)

11. cultivate a questioning attitude (1)

12. design a bespoke formal training programme for each student (1)

13. discuss options early on; discuss the 'research question' and the importance of its specificity; agree on research questions and protocol several weeks before start of project period (5)

14. early planning; plan well ahead for student (4)

15. encourage student to develop skills in minute taking during student-supervisor meeting and larger research team meetings (1)

16. encourage student to interact with patient and family (1)

[1] Numbers in parentheses indicate number of supervisor quotations corresponding to given codes.
[2] Parentheses have been used here to highlight the fact that while it is was not made explicit in the original response that this action was intended specifically for the student, this interpretation has been assumed as the one which is most likely to have been originally intended.

17. ensure a point of contact will be available on a daily basis; advise the student on the main contact; identify key player from team as point of contact, including when obtaining data (4)

18. ensure that introduced to everyone at departmental meetings, not just team members (1)

19. ensure that the student is clear about the value which their contribution could make to current knowledge and practise on a wider scale (1)

20. explain possible obstacles and contradictions (1)

21. familiarise yourself with the student's timetable at an individual level (1)

22. fulfil your personal responsibility of spending time with your student (1)

23. get involved in the project; be actively involved throughout (4)

24. get student involved in prospective study (2)

25. get student started prior to the commencement of the project period; try to get student involved early on (3)

26. get student to present their findings; student presenting data; student presenting their research at one of your group seminars; discuss deliverables, such as presentation, which might enhance career at outset; encourage submissions for high profile meetings, where applicants must compete (5)

27. get student to sketch out their final report before they start (1)

28. give them a good introductory book on research methods (1)

29. give them an introduction to the topic (2)

30. have clear goals from the outset; set clear outcomes; have clear objectives; ensure project is clear (4)

31. have student shadow team member (1)

32. have student spend time over coffee or lunch or similar with team member(s); arrange for student to have informal discussions with team members (3)

33. identify the individualized learning needs of each student; recognize that all students equally challenging (1)

34. include other team members in review meetings; meet regularly as a team (2)

35. include persons who have already been involved in similar projects in the planning process; ensure they meet such persons (2)

36. inform of previous successes (1)

37. introduce personally to all team members; get them involved with the clinical activities of the team (8)

38. introduce to team early on; get them involved with the team from an early stage (5)

39. involve clinical research fellows; involve junior doctor with interest in topic (1)

40. involve previous student as mentor or tutor to new student in large ongoing project (3)

41. make yourself accessible; take into account absence of research experience by allowing lots of time in your schedule to provide guidance (2)

42. meet regularly to review progress and short-term goals; request regular updates (2)

43. plan for funding - presentation at national or international meeting; seek to remove potential logistical problems prior to start (2)

44. plan project to ensure early successes based on prior experience; set or agree on realistic goals (4)

45. prior experience of lab research or relevant intercalated honours experience highly desirable for lab-based projects (2)

46. put in touch with all specialists for all necessary training; encourage student to sign up for relevant courses (2)

47. shadowing your student at discussion and planning meetings (1)

48. student attendance at: lab meetings, departmental meetings, relevant research seminars and clinical meetings, multi-disciplinary team meetings, clinical rounds and meetings, clinical sessions run by your colleagues (8)

49. tailor project in line with research interests of own group (4)

50. take ideas seriously (1)

51. take time out at start of the project to plan or agree on organisation of time for duration of project; prepare timetable at start; ensure project feasible in time available (1)

52. with clinical projects, allow student to visit unit early on and identify any practical problems they may encounter during the project; encourage "field visits" early on (2)
