# Supplementary material for: Initiating undergraduate medical students into communities of research practise: what do supervisors recommend?
Source: BMC Med Educ. 2010 Nov 19;10:83. doi: 10.1186/1472-6920-10-83 (PMC2998509; doi:10.1186/1472-6920-10-83)
Supplement: Additional file 2 — Appendix B. 7 Overarching themes for good supervisory practise and associated coding frame [file 1472-6920-10-83-S2.PDF]

Appendix B

7 Overarching themes for good supervisory practise and associated coding frame

Note. Codes are included in square parentheses within each theme which they have been taken to fall under. Each code is in turn highlighted in bold exclusively under the corresponding theme which was considered to be the most compelling one.

Planning the research for the student

Codes (17): **[be on the lookout for potential problems which the student may encounter with notes reviews]** [consult statistician early on] **[discuss options early on; discuss the 'research question' and the importance of its specificity; agree on research questions and protocol several weeks before start of project period] [early planning; plan well ahead for student]** [ensure a point of contact will be available on a daily basis; advise the student on the main contact; identify key player from team as point of contact, including when obtaining data] [familiarise yourself with the student's timetable at an individual level] **[get student involved in prospective study] [have clear goals from the outset; set clear outcomes; have clear objectives; ensure project is clear]** [include persons who have already been involved in similar projects in the planning process; ensure they meet such persons] [involve clinical research fellows; involve junior doctor with interest in topic] [make yourself accessible; take into account absence of research experience by allowing lots of time in your schedule to provide guidance] [meet regularly to review progress and short-term goals; request regular updates] **[plan for funding - presentation at national or international meeting; seek to remove potential logistical problems prior to start] [plan project to ensure early successes based on prior experience; set or agree on realistic goals]** [shadowing your student at discussion and planning meetings] **[tailor project in line with research interests of own group] [take time out at start of the project to plan or agree on organisation of time for duration of project; prepare timetable at start; ensure project feasible in time available]**

Quotations: 42

Preparing the student for the research

Codes total (21)

Clearly defining roles, rights of ownership and expectations
Codes (7): **[brief student on what is expected during the attachment; clarify the student's role within the team; explain the role of the researcher in a lab setting] [clarify rights of ownership of data and role in preparing work for publication]** [consult statistician early on] [ensure that the student is clear about the value which their contribution could make to current knowledge and practise on a wider scale] **[explain possible obstacles and contradictions]** [get student to present their findings; student presenting data; students presenting their research at one of your group seminars; discuss deliverables, such as presentation, which might enhance career at outset; encourage submissions for high profile meetings, where applicants must compete] [get student to sketch out their final report before they start]

Getting to know the student
Codes (4): **[design a bespoke formal training programme for each student] [familiarise yourself with the student's timetable at an individual level] [identify the individualized learning needs of each student; recognize that all students equally challenging]** [make yourself accessible; take into account absence of research **experience by allowing lots of time in your schedule to provide guidance]**

Engaging the student in prior learning
Codes (9): [(get student to)[1] read reports of previous students] **[allow plenty of lead time to consider ideas and carry out background reading; allow time prior to project period for background reading] [allow the student to immerse themselves in the topic by getting them to carry out a literature review prior to practising basic techniques]** [consult statistician early on] **[give them a good introductory book on research methods] [give them an introduction to the topic] [prior experience of lab research or relevant intercalated honours experience highly desirable for lab-based projects] [put in touch with all specialists for all necessary training; encourage student to sign up for relevant courses] [with clinical projects, allow student to visit unit early on and identify any practical problems they may encounter during the project; encourage "field visits" early on]**

Getting the student started
Codes (1): **[get student started prior to the commencement of the project period; try to get student involved early on]**

Quotations: 40

[1] Parentheses have been used here to highlight the fact that while it is was not made explicit in the original response that this action was intended specifically for the student, this interpretation has been assumed as the one which is most likely to have been originally intended.

Connecting the student with others

Codes (19): [(get student to)[1] read reports of previous students] [Advise them of departmental meetings beyond the scope of their project] **[allocate room with other students, including postgraduate research students; involve junior postgraduate colleagues] [consult statistician early on] [encourage student to interact with patient and family] [ensure a point of contact will be available on a daily basis; advise the student on the main contact; identify key player from team as point of contact, including when obtaining data] [ensure that introduced to everyone at departmental meetings, not just team members]** [get student to present their findings; student presenting data; student presenting their research at one of your group seminars; discuss deliverables, such as presentation, which might enhance career at outset; encourage submissions for high profile meetings, where applicants must compete] **[have student shadow team member] [have student spend time over coffee or lunch or similar with team member(s); arrange for student to have informal discussions with team members]** [include other team members in review meetings; meet regularly as a team] **[include persons who have already been involved in similar projects in the planning process; ensure they meet such persons] [introduce personally to all team members; get them involved with the clinical activities of the team] [introduce to team early on; get them involved with the team from an early stage] [involve clinical research fellows; involve junior doctor with interest in topic] [involve previous student as mentor or tutor to new student in large ongoing project]** [put in touch with all specialists for all necessary training; encourage student to sign up for relevant courses] [student attendance at: lab meetings, departmental meetings, relevant research seminars and clinical meetings, multi-disciplinary team meetings, clinical rounds and meetings, clinical sessions run by your colleagues] [tailor project in line with research interests of own group]

Quotations: 57

[1] Parentheses have been used here to highlight the fact that while it is was not made explicit in the original response that this action was intended specifically for the student, this interpretation has been assumed as the one which is most likely to have been originally intended.

Cultivating a sense of accountability

Codes (17): [allocate room with other students, including postgraduate research students; involve junior postgraduate colleagues] [allow plenty of lead time to consider ideas and carry out background reading; allow time prior to project period for background reading] [brief student on what is expected during the attachment; clarify the student's role within the team; explain the role of the researcher in a lab setting] [consult statistician early on] **[encourage student to develop skills in minute taking during student-supervisor meeting and larger research team meetings]** [ensure that the student is clear about the value which their contribution could make to current knowledge and practise on a wider scale] [get student involved in prospective study] [get student to present their findings; student presenting data; students presenting their research at one of your group seminars; discuss deliverables, such as presentation, which might enhance career at outset; encourage submissions for high profile meetings, where applicants must compete] [get student to sketch out their final report before they start] [give them a good introductory book on research methods] [include other team members in review meetings; meet regularly as a team] [introduce to team early on; get them involved with the team from an early stage] [involve clinical research fellows; involve junior doctor with interest in topic] **[meet regularly to review progress and short-term goals; request regular updates]** [put in touch with all specialists for all necessary training; encourage student to sign up for relevant courses] **[shadowing your student at discussion and planning meetings]** [tailor project in line with research interests of own group]

Quotations: 41

Fostering a holistic perspective of the subject area(s)

Codes (10): [(get student to)[1] read reports of previous students] **[advise them of departmental meetings beyond the scope of their project]** [allow the student to immerse themselves in the topic by getting them to carry out a literature review prior to practising basic techniques] [encourage student to develop skills in minute taking during student-supervisor meeting and larger research team meetings] [ensure that introduced to everyone at departmental meetings, not just team members] **[ensure that the student is clear about the value which their contribution could make to current knowledge and practise on a wider scale]** [get student to present their findings; student presenting data; students presenting their research at one of your group seminars; discuss deliverables, such as presentation, which might enhance career at outset; encourage submissions for high profile meetings, where applicants must compete] [give them an introduction to the topic] [have student shadow team member] **[student attendance at: lab meetings, departmental meetings, relevant research seminars and clinical meetings, multi-disciplinary team meetings, clinical rounds and meetings, clinical sessions run by your colleagues]**

Quotations: 24

[1] Parentheses have been used here to highlight the fact that while it is was not made explicit in the original response that this action was intended specifically for the student, this interpretation has been assumed as the one which is most likely to have been originally intended.

Cultivating self-efficacy in research competence

Codes (21): [**(get student to)**[1] **read reports of previous students**] [allow plenty of lead time to consider ideas and carry out background reading; allow time prior to project period for background reading] [allow the student to immerse themselves in the topic by getting them to carry out a literature review prior to practising basic techniques] **[be enthusiastic about project or subject area, or something else]** [clarify rights of ownership of data and role in preparing work for publication] **[cultivate a questioning attitude]** [discuss options early on; discuss the 'research question' and the importance of its specificity; agree on research questions and protocol several weeks before start of project period] [encourage student to interact with patient and family] [ensure that the student is clear about the value which their contribution could make to current knowledge and practise on a wider scale] [explain possible obstacles and contradictions] [fulfil your personal responsibility of spending time with your student] **[get student to present their findings; student presenting data; student presenting their research at one of your group seminars; discuss deliverables, such as presentation, which might enhance career at outset; encourage submissions for high profile meetings, where applicants must compete]** [give them a good introductory book on research methods] [give them an introduction to the topic] **[inform of previous successes]** [make yourself accessible; take time out at start of the project to plan or agree on organisation of time for duration of project; prepare timetable at start; ensure project feasible in time available] [plan project to ensure early successes based on prior experience; set or agree on realistic goals] [prior experience of lab research or relevant intercalated honours experience highly desirable for lab-based projects] [put in touch with all specialists for all necessary training; encourage student to sign up for relevant courses] [tailor project in line with research interests of own group] **[take ideas seriously]**

Quotations: 46

[1] Parentheses have been used here to highlight the fact that while it is was not made explicit in the original response that this action was intended specifically for the student, this interpretation has been assumed as the one which is most likely to have been originally intended.

Working with the student

Codes (15): [be on the lookout for potential problems which the student may encounter with notes reviews] [consult statistician early on] [cultivate a questioning attitude] [discuss options early on; discuss the 'research question' and the importance of its specificity; agree on research questions and protocol several weeks before start of project period] **[fulfil your personal responsibility of spending time with your student] [get involved in the project; be actively involved throughout]** [get student started prior to the commencement of the project period; try to get student involved early on] **[get student to sketch out their final report before they start]** [give them an introduction to the topic] [introduce to team early on; get them involved with the team from an early stage] **[make yourself accessible; take into account absence of research experience by allowing lots of time in your schedule to provide guidance]** [meet regularly to review progress and short-term goals; request regular updates] [shadowing your student at discussion and planning meetings] [take ideas seriously] [take time out at start of the project to plan or agree on organisation of time for duration of project; prepare timetable at start; ensure project feasible in time available]

Quotations: 31
